# Supplementary material for: Indirect H2O2 synthesis without H2
Source: Nat Commun. 2024 Jan 26;15:766. doi: 10.1038/s41467-024-44741-1 (PMC10817937; doi:10.1038/s41467-024-44741-1)
Supplement: Supplementary file 1 — Supplementary Information [file 41467_2024_44741_MOESM1_ESM.pdf]

Supporting Information for:

Indirect H_2O_2 synthesis without H_2

Arthur G. Fink,[1] Roxanna S. Delima,[2,3] Alexandra R. Rousseau,[2,3] Camden Hunt,[1,2] Natalie E. LeSage,[1] Aoxue Huang,[1] Monika Stolar,[1] and Curtis P. Berlinguette[1,2,3,4,]*

[1]Department of Chemistry, The University of British Columbia, 2036 Main Mall, Vancouver, British Columbia, V6T 1Z1, Canada.

[2]Stewart Blusson Quantum Matter Institute, The University of British Columbia, 2355 East Mall, Vancouver, British Columbia, V6T 1Z4, Canada.

[3]Department of Chemical and Biological Engineering, The University of British Columbia, 2360 East Mall, Vancouver, British Columbia, V6T 1Z3, Canada.

[4]Canadian Institute for Advanced Research (CIFAR), 661 University Avenue, Toronto, Ontario, M5G 1M1, Canada.

*Corresponding author. Curtis P. Berlinguette. Email: cberling@chem.ubc.ca.

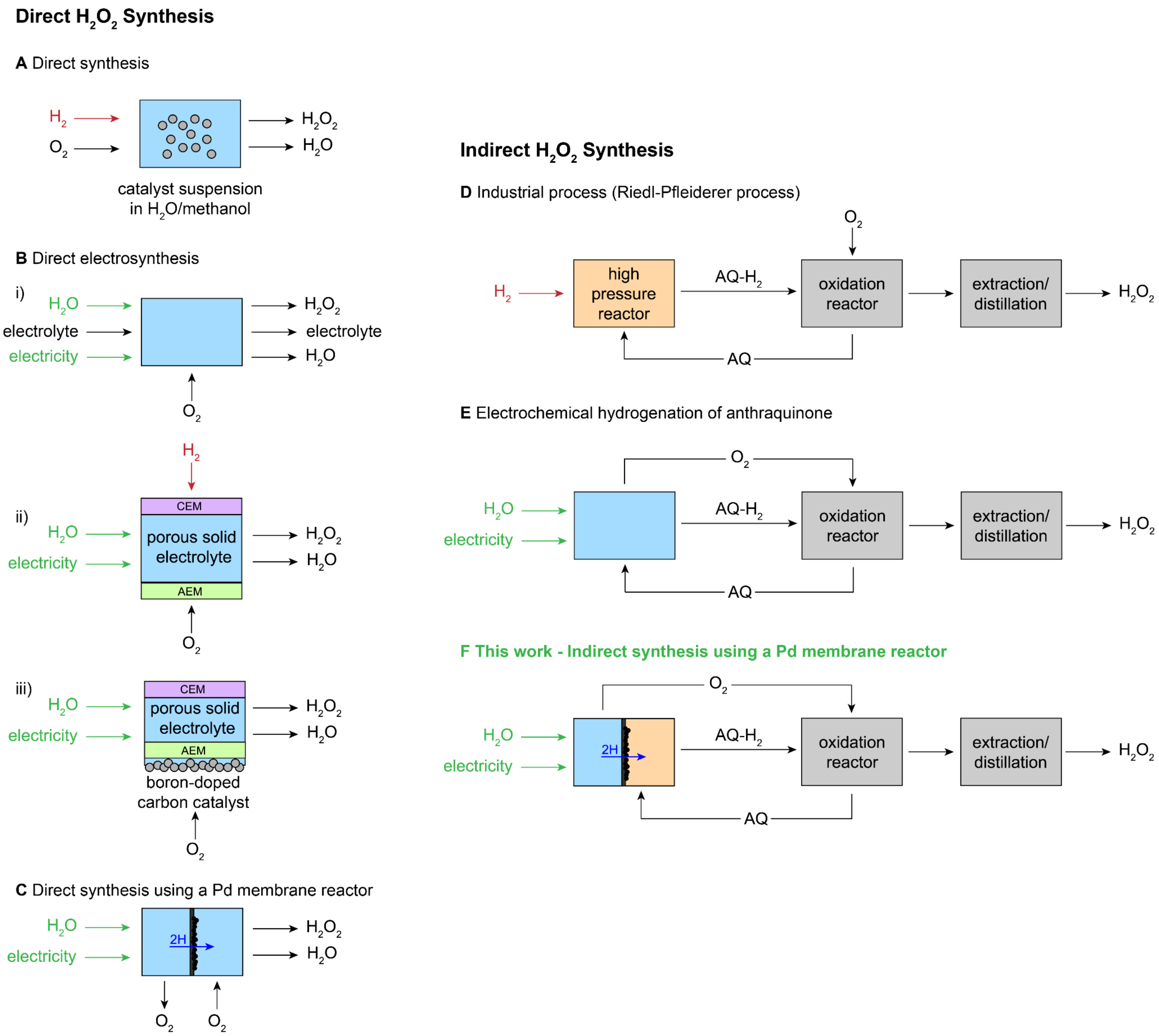

Supplementary Fig. 1 | Schematic of hydrogen peroxide (H_2O_2) synthesis by direct and indirect methods; (**A**) Direct synthesis requires the presence of both H_2 and O_2 gases in a single reactor at high pressure over a catalyst suspension[1]; (**B**) Direct electrochemical H_2O_2 synthesis can proceed through the following methods: (i) water is oxidized at the anode in the presence of an electrolyte and reacts with O_2 at the cathode to generate H_2O_2 [2]; (ii) H_2 gas is oxidized to H^+ at the anode and reacts with O_2 at the cathode to generate H_2O_2 [3]; or (iii) water is oxidized at the anode and reacts with O_2, that is reduced on a boron-doped cathode, to generate H_2O_2 [4]; (**C**) Direct synthesis using a Pd membrane reactor proceeds when hydrogen atoms are produced from water electrolysis, occuring in a separate reactor compartment from the production of H_2O_2 [5]. The hydrogen atoms move through the membrane where they react with

O_2 to form H_2O_2; (**D**) The indirect industrial process proceeds by anthraquinone (AQ) hydrogenation using H_2 gas to form anthraquinol (AQ-H_2)[6]. Subsequent oxidation of anthraquinol (in a separate rector) reforms the original anthraquinone in tandem with H_2O_2 formation. This is known as the Riedl-Pfleiderer process; (**E**) Electrochemical hydrogenation of anthraquinone to produce H_2O_2 proceeds when anthraquinone is used as a redox mediator in an aqueous solution, transferring hydrogen produced from water electrolysis to O_2 [7]. (**F**) Indirect synthesis using the Pd membrane reactor proceeds when hydrogen is sourced from water electrolysis and separated from the AQ by the Pd membrane. This allows for efficient water electrolysis conditions in one compartment of the reactor while maintaining the organic solution in the other compartment of the reactor. This process allows for direct integration with current industrial methods for H_2O_2 production. Hydrogen gas denoted in red represents hydrogen sourced from steam-methane reforming. Water and electricity denoted in green represent inputs that can be sourced from renewable energy. Reactor color code: blue = aqueous; orange = organic; purple = cation exchange membrane (CEM); green = anion exchange membrane (AEM).

Supplementary Table 1: Key experimental conditions for direct and indirect H_2O_2 production.

	Direct					Indirect		
	Synthesis[1]	Electrosynthesis			Palladium membrane reactor (previous work)[5]	Industrial process[6]	Electrochemical[7]	Palladium membrane reactor (this work)
		i. (Yamanaka *et al.*)[2]	ii. (Wang *et al.*)[3]	iii. (Wang *et al.*)[4]				
**H source**	$H_{2(g)}$	H_2O	$H_{2(g)}$	H_2O	H_2O	$H_{2(g)}$	H_2O	H_2O
**Pressure**	5–100 atm	ambient	ambient	ambient	ambient	1.5–3.5 atm	ambient	ambient
**Temperature**	ambient	ambient	ambient	ambient	ambient	45 ºC	ambient	60 ºC
**Solvent**	acidic aqueous or acidic alcohol	alkaline aqueous	H_2O	H_2O	H_2O/ methanol	organic	H_2O	flexible
**Current density**	-	100 mA cm^{-2}	200 mA cm^{-2}	400 mA cm^{-2}	≤100 mA cm^{-2}	-	10 mA cm^{-2}	≤150 mA cm^{-2}
**Current efficiency**	-	93%	22.6%	85%	9.6% @10 mA cm^{-2}<br><br>1.8% @100 mA cm^{-2}	-	80%	80±7% @75 mA cm^{-2}<br><br>42±4% @150 mA cm^{-2}
**H_2O_2 production**	-	2.12 g L^{-1}	6.29 g L^{-1}	1.1 g L^{-1} @30 mA cm^{-2}	0.122 g L^{-1} @10 mA cm^{-2}<br><br>0.233 g L^{-1} @100 mA cm^{-2}	≤26.42 g L^{-1} (after distillation)	1.12 g L^{-1}	5.24 g L^{-1} @75 mA cm^{-2}

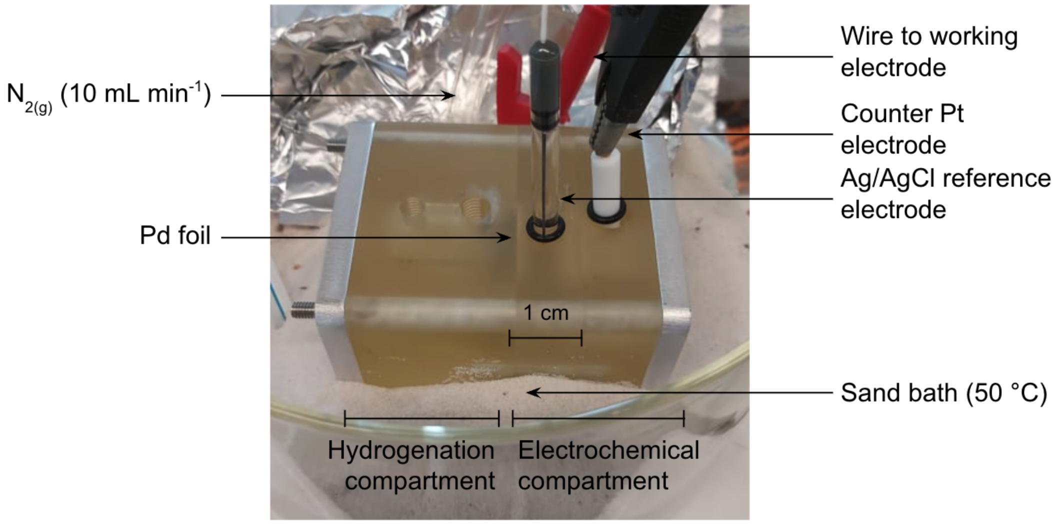

Supplementary Fig. 2 | Batch reactor setup showing wires to the working electrode, counter Pt electrode, Ag/AgCl reference electrode, Pd foil position, $N_{2(g)}$ inlet, heating sand bath, electrochemical chamber, and hydrogenation chamber.

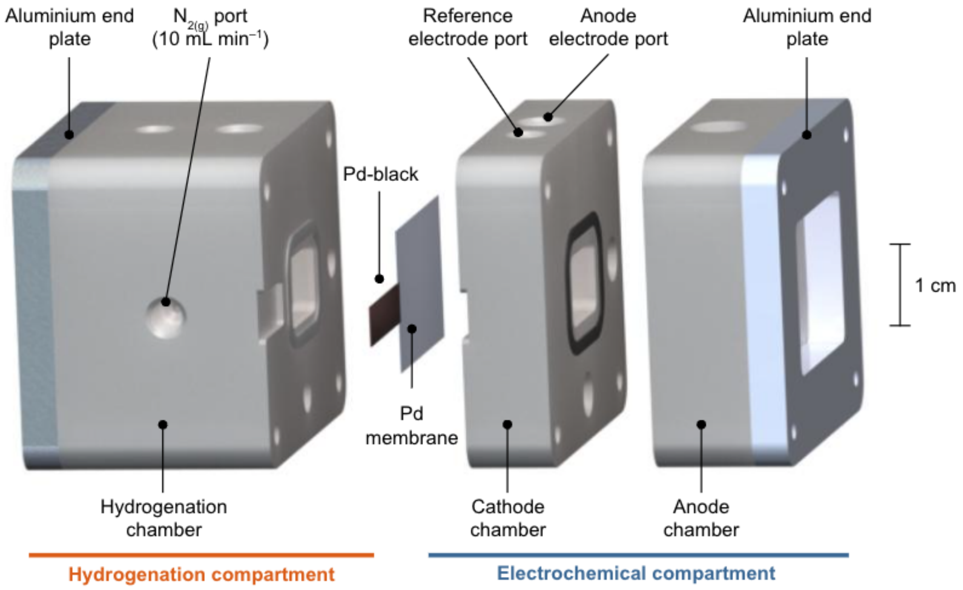

Supplementary Fig. 3 | CAD design of the batch reactor. The 2-compartment cell contains an electrochemical compartment filled with 8 mL of 1 M H_2SO_4 and a hydrogenation compartment filled with 0.25 M AQ in 8 mL of a mixture of xylenes:DIBC 1:1 *v/v*. The electrochemical compartment contains a platinum anode, a Ag/AgCl reference electrode, and the exposed Pd membrane surface. Catalyst (electrodeposited Pd) is coated on the side of the membrane exposed to the hydrogenation compartment.

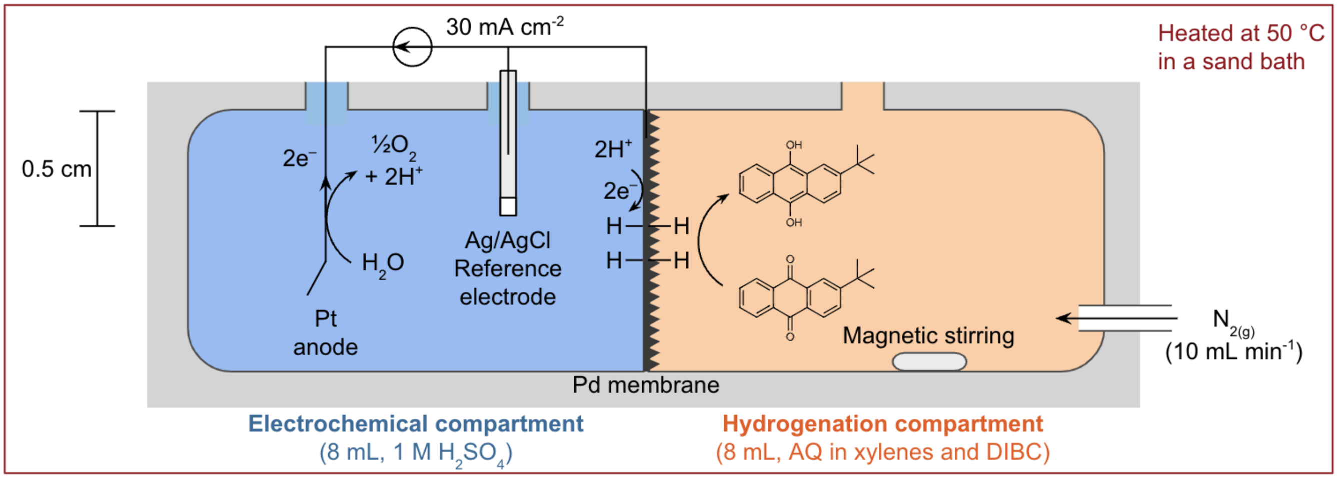

Supplementary Fig. 4 | Schematic representation of the batch reactor. The batch reactor consists of an electrochemical compartment (blue) and a hydrogenation compartment (orange) that sandwich the Pd membrane. The electrochemical compartment contains 8 mL of 1 M H_2SO_4 and is fitted with a Pt counter electrode and a Ag/AgCl reference electrode. The hydrogenation compartment contains 8 mL of the hydrogenation solution (250 mM 2-*tert*-butylanthraquinone (AQ) in a mixture of xylenes:DIBC 1:1 (*v/v*)). $N_{2(g)}$ is supplied to the hydrogenation compartment at 10 mL min^{-1}. The batch reactor was heated at the desired temperature by means of a sand bath. The temperature was measured in the electrochemical compartment.

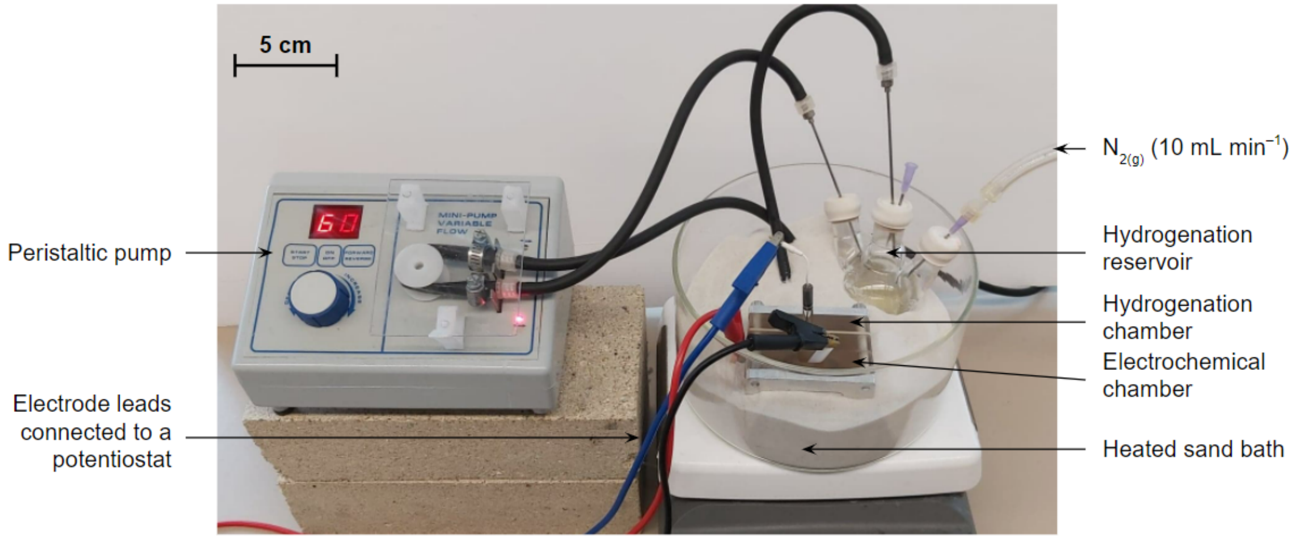

Supplementary Fig. 5 | Flow reactor setup showing electrode leads connected to a potentiostat (not shown), the flow reactor consisting of the hydrogenation chamber and the electrochemical chamber, the hydrogenation reservoir, the heated sand bath, the $N_{2(g)}$ inlet, and the peristaltic pump.

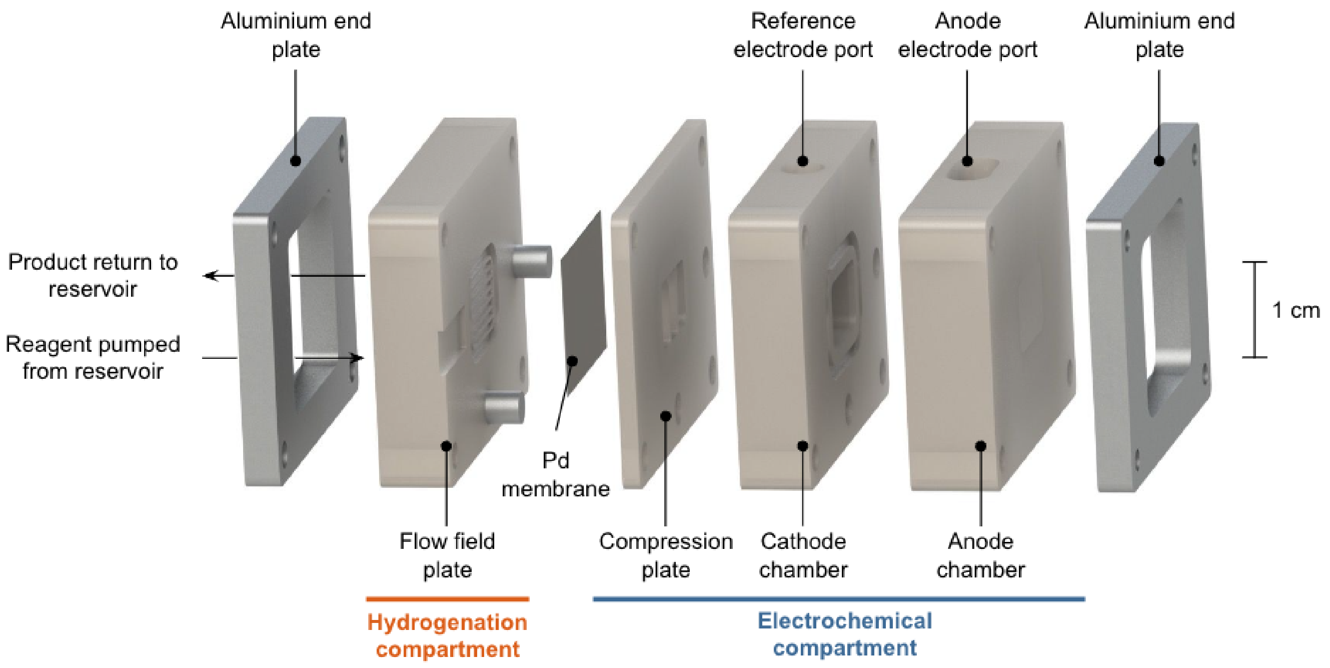

Supplementary Fig. 6 | CAD design of the flow reactor. The 2-compartment cell contains an electrochemical compartment filled with 8 mL of 1 M H_2SO_4 and a hydrogenation compartment that has a flow field plate where the hydrogenation solution is passed. The electrochemical compartment contains a platinum anode, a Ag/AgCl reference electrode, and the exposed Pd membrane surface. Catalyst (electrodeposited Pd-black, not shown) is coated on the side of the membrane exposed to the hydrogenation compartment.

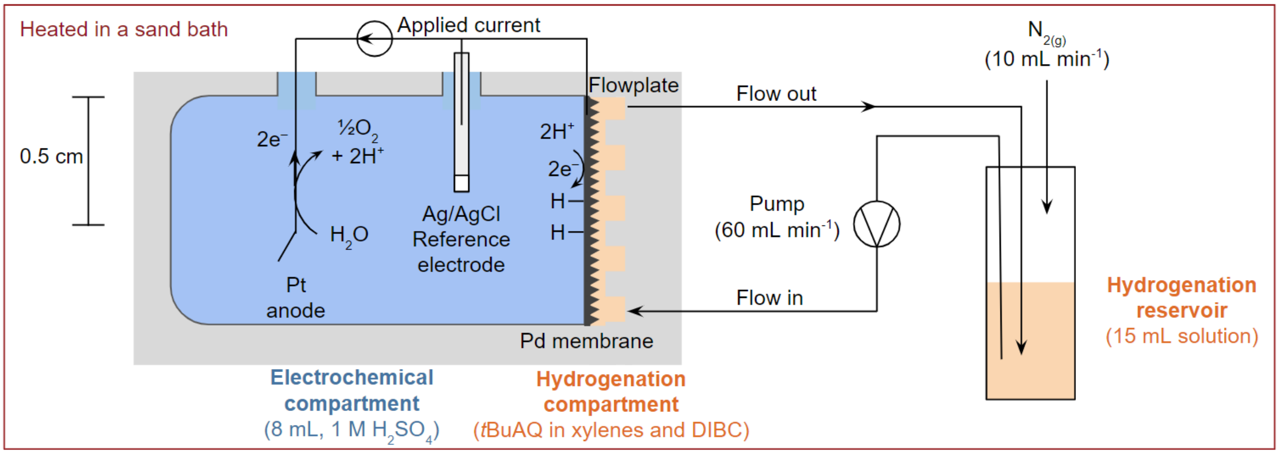

Supplementary Fig. 7 | Schematic representation of the flow reactor and setup. The flow reactor consists of an electrochemical compartment (blue) and a hydrogenation compartment (orange) that sandwich the Pd membrane. The electrochemical compartment contains 8 mL of 1 M H_2SO_4 and is fitted with a Pt counter electrode and a Ag/AgCl reference electrode. The hydrogenation compartment is continuously fed with 15 mL of the hydrogenation solution (250 mM 2-*tert*-butylanthraquinone (AQ) in a mixture of xylenes and DIBC) by means of a peristaltic pump at 60 mL min^{-1}. The headspace of the hydrogenation reservoir is continuously fed with $N_{2(g)}$ at 10 mL min^{-1}. The flow reactor and the hydrogenation reservoir were heated at the desired temperature by means of a sand bath. The temperature was measured in the electrochemical compartment.

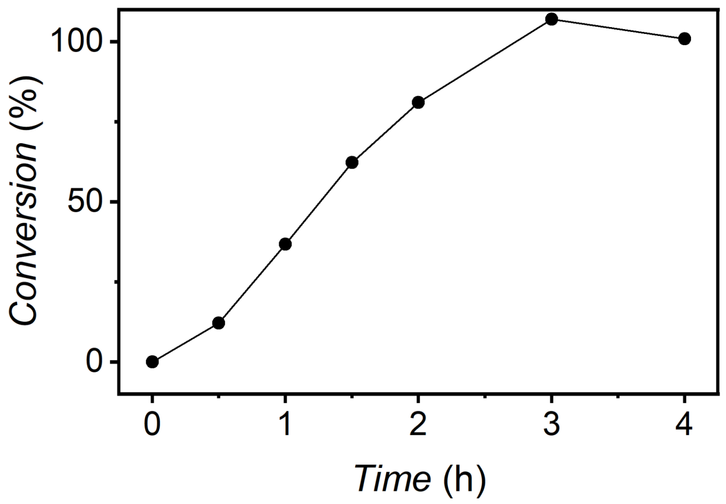

Supplementary Fig. 8 | Conversion of AQ versus time of reaction as quantified by iodometric titrations during electrolysis at 30 mA cm^{-2} in the batch reactor.

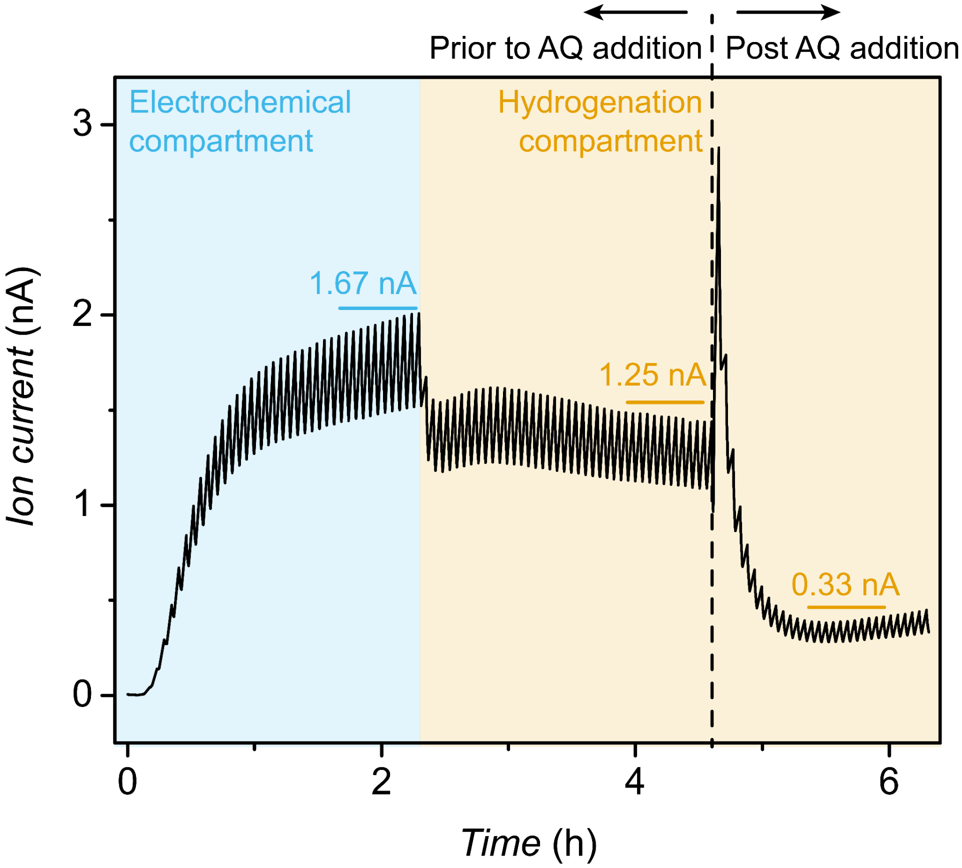

Supplementary Fig. 9 | Continuous *in-situ* atmospheric–mass spectrometry in a batch cell at 50 °C and 75 mA cm^{-2}. In blue is the H_2 evolving in the electrochemical compartment. In orange is the H_2 evolving in the hydrogenation compartment. The dashed line denotes the time at which anthraquinone (AQ) was added into the hydrogenation chamber. This graph shows 43% of the hydrogen produced goes through the dense Pd membrane to undergo the hydrogen evolution reaction in the hydrogenation compartment. At 4.6 hours, the hydrogenation compartment is spiked with *tert*-butylanthraquinone to reach 0.25 M. The sharp decrease in hydrogen evolution is caused by the consumption of hydrogen in the Pd-catalyzed hydrogenation reaction of AQ. The calculated current efficiency for anthraquinone hydrogenation from this atmospheric–mass spectrometry data is 30%. Results were plotted over time only to show the stability of the signal.

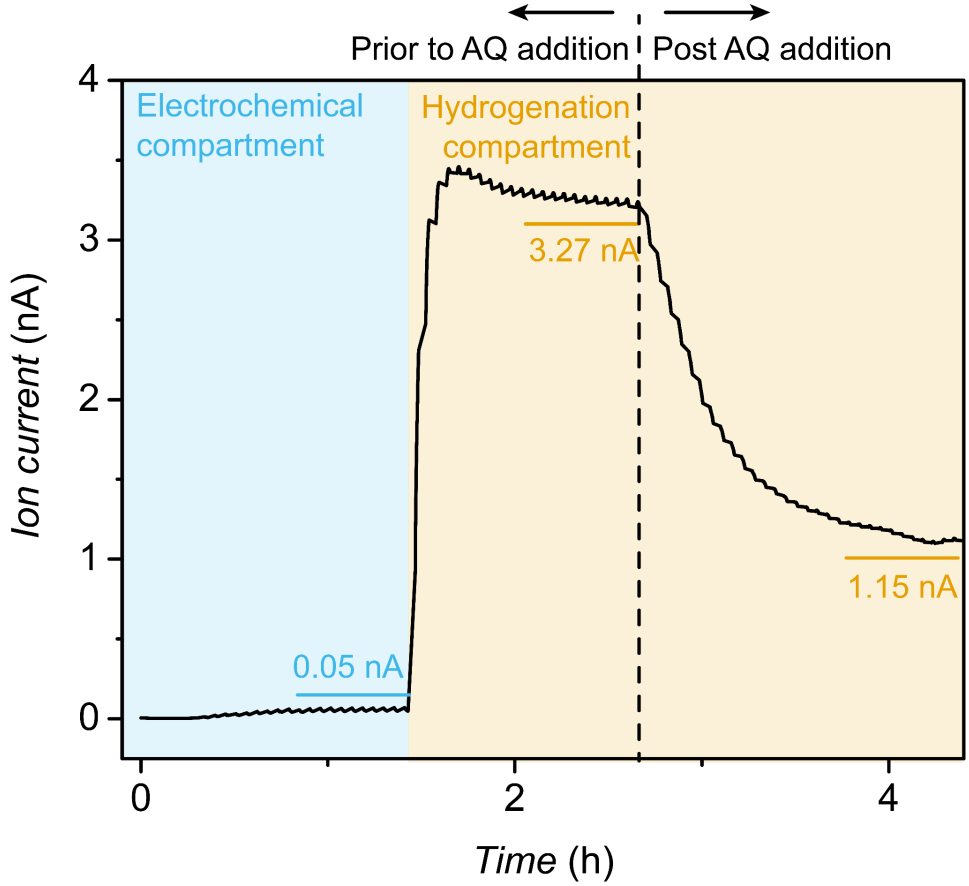

Supplementary Fig. 10 | Continuous *in-situ* atmospheric–mass spectrometry in a flow cell at 50 °C and 75 mA cm^{-2}. In blue is the H_2 evolving in the electrochemical compartment. In orange is the H_2 evolving in the hydrogenation compartment. The dashed line denotes the time at which anthraquinone (AQ) was added into the hydrogenation chamber. This graph shows 99% of the hydrogen produced goes through the dense Pd membrane to undergo the hydrogen evolution reaction in the hydrogenation compartment. At 2.75 hours, the hydrogenation compartment is spiked with *tert*-butylanthraquinone to reach 0.25 M. The sharp decrease in hydrogen evolution is caused by the consumption of hydrogen in the Pd-catalyzed hydrogenation reaction of AQ. The calculated current efficiency for anthraquinone hydrogenation from this atmospheric–mass spectrometry data is 64%. Results were plotted over time only to show the stability of the signal.

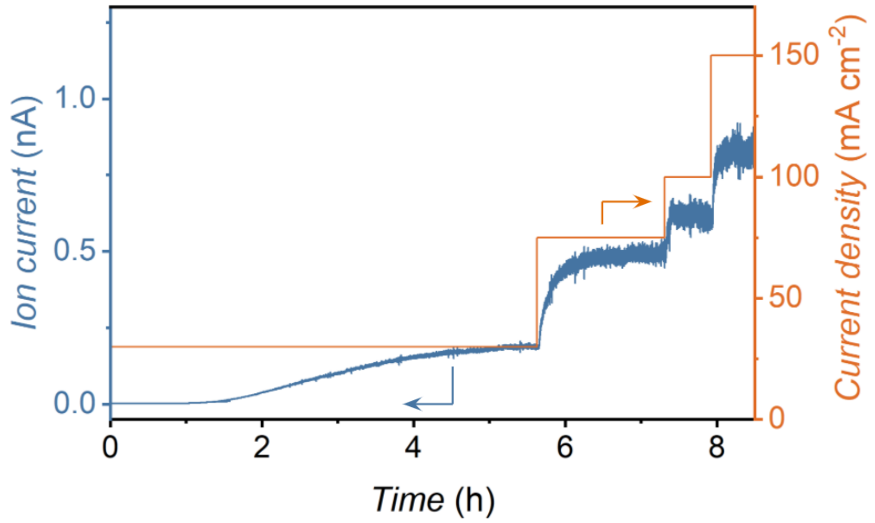

Supplementary Fig. 11 | Graph of ion current (left y-axis, blue) versus time and applied current density (right y-axis, orange) versus time for a continuous electrolysis experiment measured in the hydrogenation compartment of the flow reactor at 60 ºC. The ion current is proportional to H_2 permeation through the Pd foil membrane as measured by atmospheric–mass spectrometry.

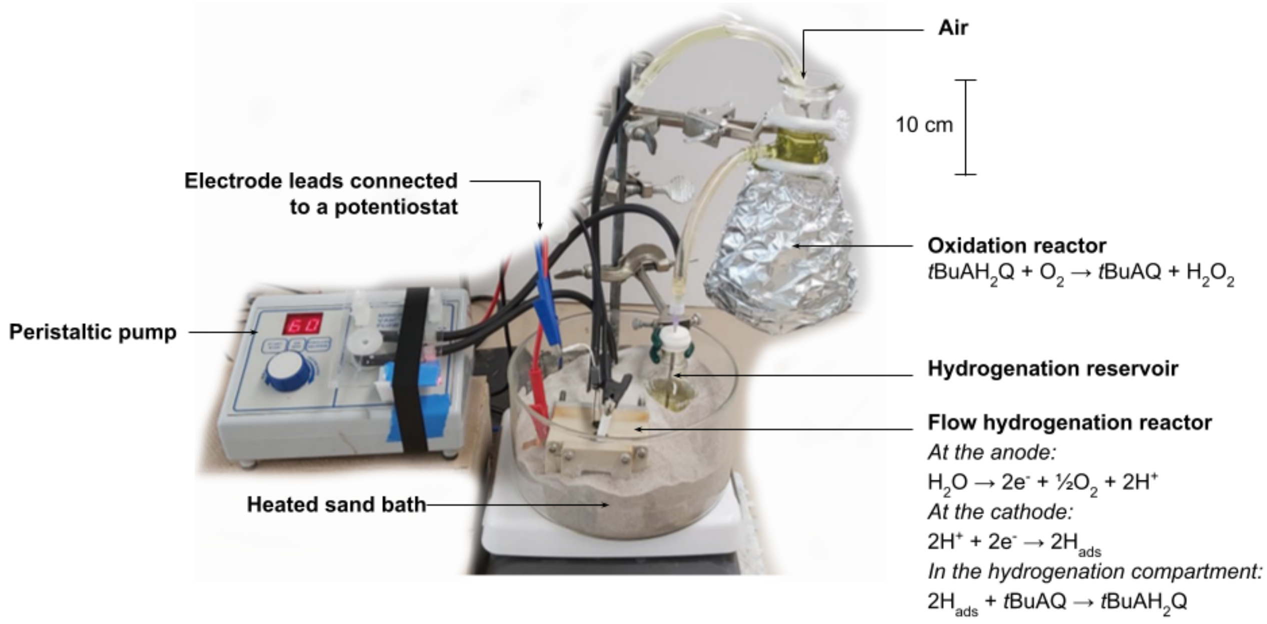

Supplementary Fig. 12 | Flow reactor and oxidation reactor setup showing the flow hydrogenation reactor, the oxidation reactor, the hydrogenation reservoir, the electrode leads connected to a potentiostat (not shown), the heated sand bath, the peristaltic pump, and the input of air to the oxidation reactor.

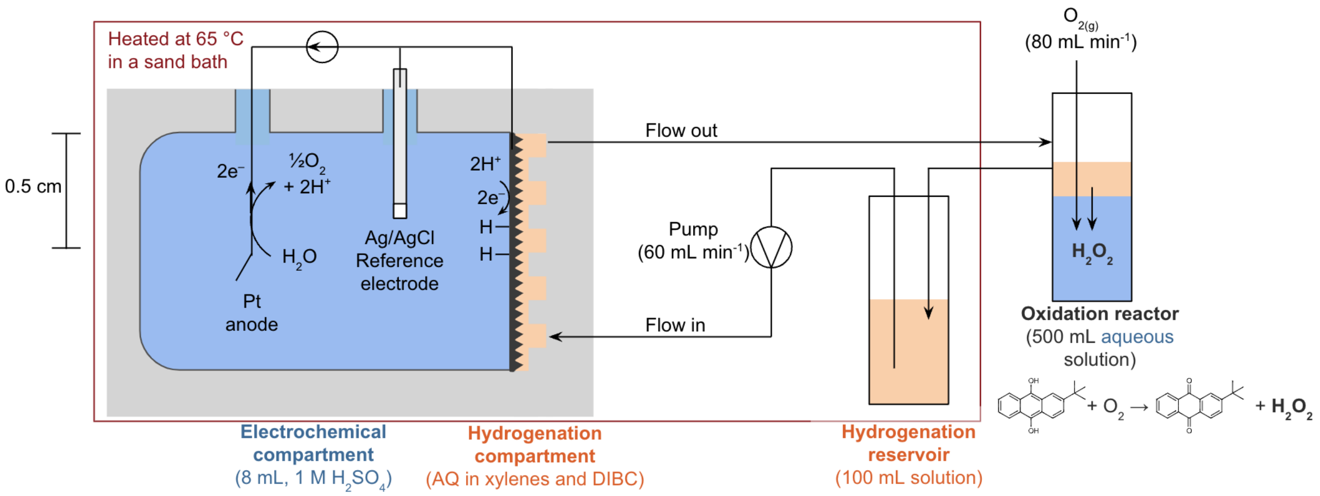

Supplementary Fig. 13 | Schematic representation of the setup for H_2O_2 production. The flow cell is operated as described above but with a hydrogenation solution of 100 mL total that is fed to the flow reactor from a hydrogenation solution reservoir. The hydrogenation solution exiting the flow reactor is then fed to an oxidation reactor containing 500 mL of H_2O containing 1 mM EDTA and 3 mM citric acid stabilizers (in blue; denser than the organic solution containing the anthraquinone represented here in orange) and continuously fed with air at 90 mL min^{-1}. In the oxidation reactor, 2-*tert*-butylanthrahydroquinone (AQ-H_2) reacts with O_2 to form H_2O_2 and 2-*tert*-butylanthraquinone (AQ) before H_2O_2 transfers into the aqueous solution. The organic hydrogenation solution is subsequently gravity-fed back to the hydrogenation reservoir for heating. Both the hydrogenation reservoir and the flow reactor are heated at 60 °C in a sand bath. The temperature was measured in the electrochemical compartment.

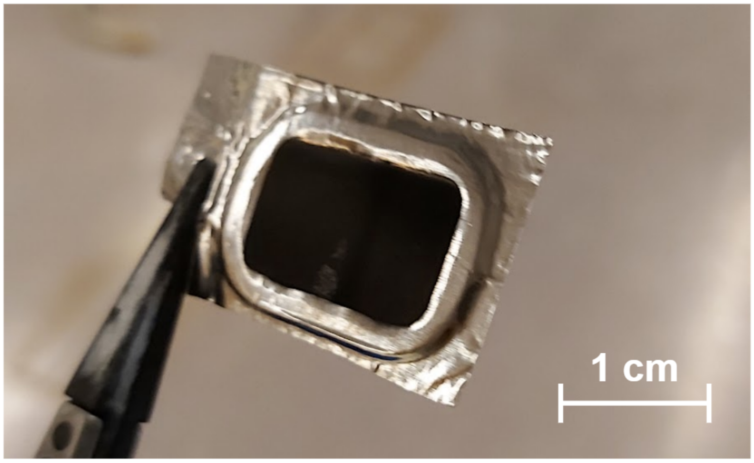

Supplementary Fig. 14 | Picture of a Pd foil with electrodeposited Pd catalyst.

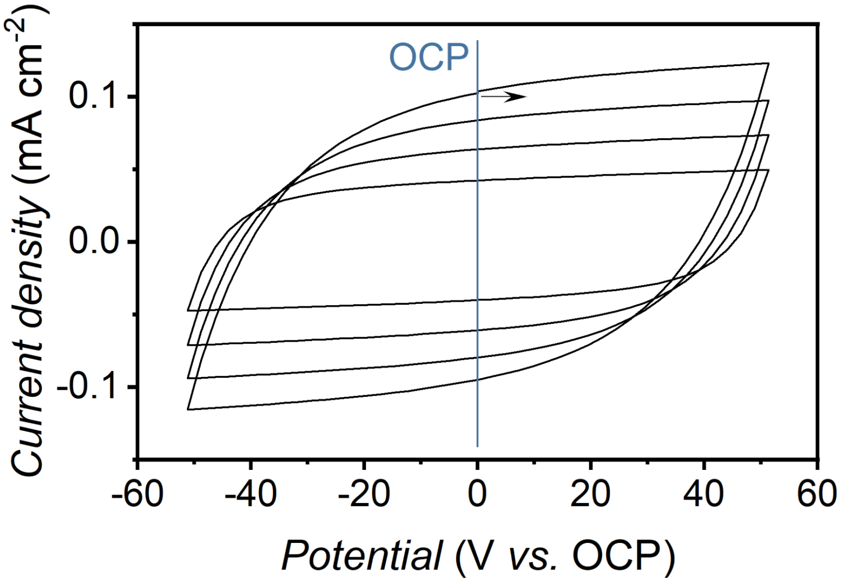

Supplementary Fig. 15 | Cyclic voltammograms (CVs) used to determine the ECSA. See the corresponding *Methods, Electrochemical active surface area measurements* section in the manuscript. Cyclic voltammograms were performed at 20, 30, 40, and 50 mA s^{-1}, over 3 cycles, and ±50 mV from the open circuit potential (OCP, identified in blue). Here only the third cycle is represented.

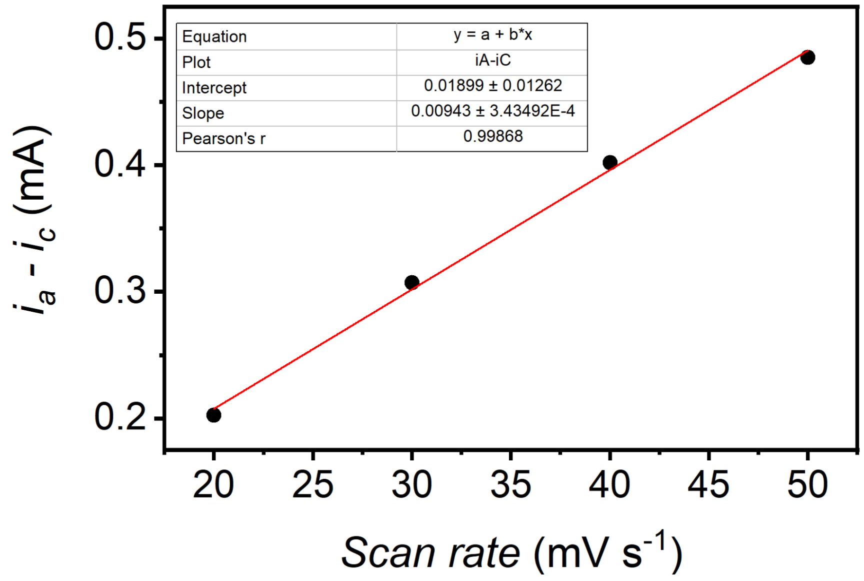

Supplementary Fig. 16 | Plot of the difference between anodic current (i_a) and cathodic current (i_c) at the OCP as determined by CVs versus scan rate of the respective CV. The data were fitted with a regression line (in red) to obtain the capacitance of the foil (i.e., the slope; 0.00943 Farads).

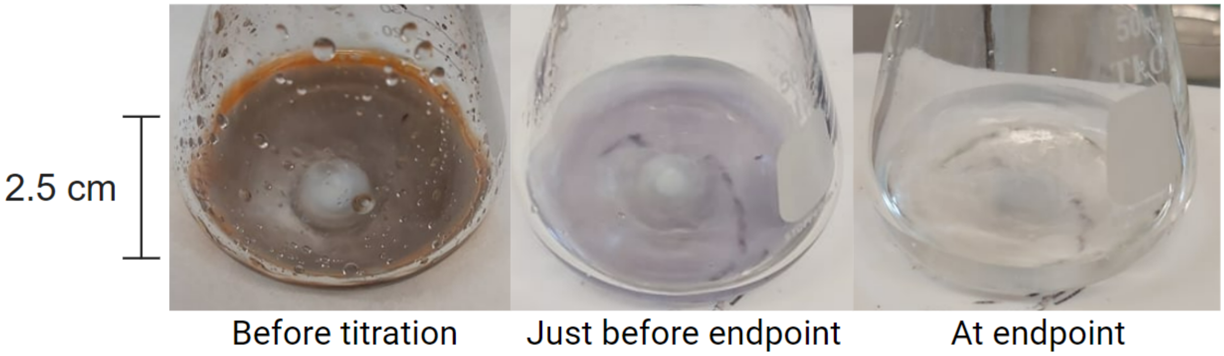

Supplementary Fig. 17 | Pictures of the various stages of iodometric titrations including just before the iodometric titration (*left*), just before the endpoint (*center*), and at the endpoint (*right*).

Supplementary Note 1 | Product identification

Although we observed 100% conversion of AQ by iodometric titration, AQ can be hydrogenated to undesired products that are not active for H_2O_2 production but could be accounted for by titration (e.g., anthrones; see Supplementary Fig. 18). We therefore performed GC–MS analyses of the hydrogenated solutions to investigate the distribution of products. The GC–MS spectrum on Supplementary Fig. 19 only presents one peak at a retention time of 11.2 min which corresponds to AQ. AQ-H_2 and H_4AQ-H_2 spontaneously react with atmospheric oxygen to form H_2O_2 or release H_2 at high temperatures >200 °C which give back AQ and H_4AQ, respectively. Anthrones (AO and H_4AO), however, are stable in air and at elevated temperatures. We did not observe such peaks corresponding to anthrones in our GC–MS analysis. Collectively, these results point to the selective generation of AQ-H_2, which is unstable under GC–MS conditions. Indeed, it was previously reported that anthrahydroquinones could not be detected by GC–MS[8].

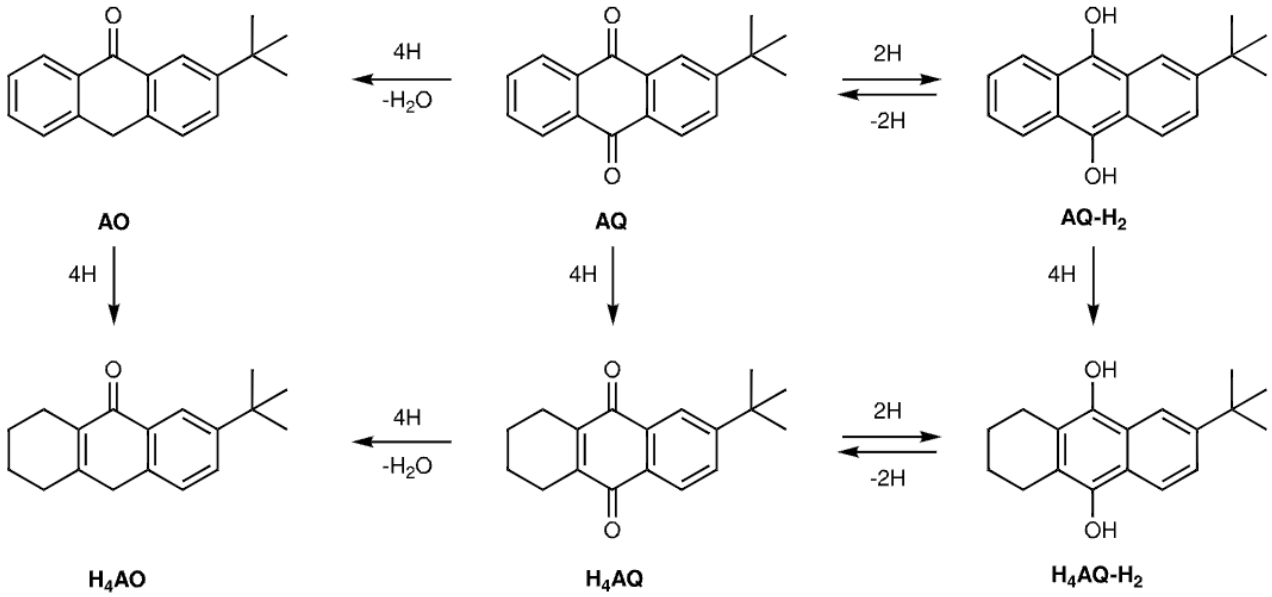

Supplementary Fig. 18 | The different possible reaction pathways of AQ hydrogenation. 2-*tert*-butylanthrone (AO) and 6-*tert*-butyl-1,2,3,4-tetrahydro-anthrone (H_4AO) are irreversibly formed and cannot be used in H_2O_2 production. 6-*tert*-butyl-1,2,3,4-tetrahydro-anthraquinone (H_4AQ) is also active for H_2O_2 production with its hydrogenated form, 6-*tert*-butyl-1,2,3,4-tetrahydro-anthrahydroquinone (H_4AQ-H_2)[8–10]. Only the peak for AQ was observed in GC–MS analyses.

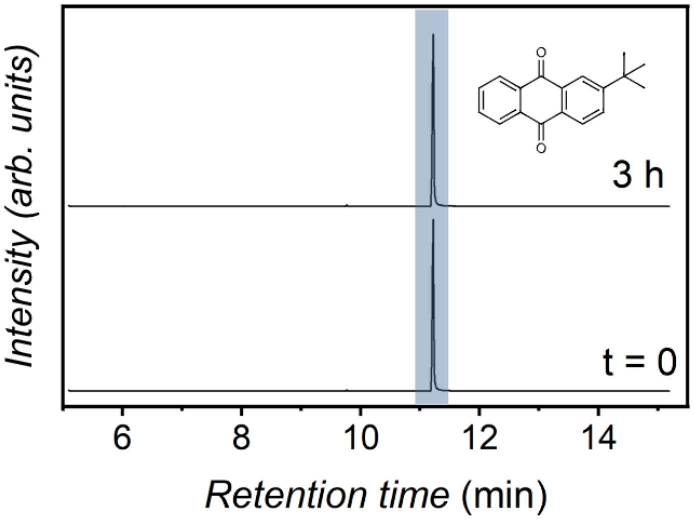

Supplementary Fig. 19 | Gas chromatograph of the GC–MS experiment displaying the intensity of the signal versus residence time. Two traces are displayed: (1) before the hydrogenation experiment (t = 0, *bottom*); and (ii) after 3 h of hydrogenation at 100 mA cm^{-2} (*top*) performed in a batch reactor at 50 ºC. The peak for AQ is identified in blue at a residence time of 11.2 min.

References

1. Flaherty, D. W. Direct synthesis of H_2O_2 from H_2 and O_2 on Pd catalysts: Current understanding, outstanding questions, and research needs. *ACS Catal.* **8**, 1520–1527 (2018).
2. Yamanaka, I., Onizawa, T., Takenaka, S. & Otsuka, K. Direct and continuous production of hydrogen peroxide with 93% selectivity using a fuel cell system. *Angew. Chem. Int. Ed.* **42**, 3653−3655 (2003).
3. Xia, C., Xia, Y., Zhu, P., Fan, L. & Wang, H. Direct electrosynthesis of pure aqueous H_2O_2 solutions up to 20% by weight using a solid electrolyte. *Science* **366**, 226–231 (2019).
4. Xia, Y., Zhao, X., Xia, C. et al. Highly active and selective oxygen reduction to H_2O_2 on boron-doped carbon for high production rates. *Nat. Commun.* **12**, 4225 (2021).
5. Huang, A. et al. Direct H_2O_2 synthesis without H_2 gas. *J. Am. Chem. Soc.* **144**, 14548–14554 (2022).
6. McDonnell, G. The use of hydrogen peroxide for disinfection and sterilization applications. in *Patai's Chemistry of Functional Groups,* 1–34 (Wiley, New York, 2014).
7. Murray, A. T., Voskian, S., Schreier, M., Hatton, T. A. & Surendranath, Y. Electrosynthesis of hydrogen peroxide by phase-transfer catalysis. *Joule* **3**, 2942–2954 (2019).
8. Drelinkiewicz, A. & Waksmundzka-Góra, A. Investigation of 2-ethylanthraquinone degradation on palladium catalysts. *J. Mol. Catal. A Chem.* **246**, 167–175 (2006).
9. Kosydar, R., Drelinkiewicz, A. & Ganhy, J. P. Degradation reactions in anthraquinone process of hydrogen peroxide synthesis. *Catal. Letters* **139**, 105–113 (2010).
10. Liu, B., Qiao, M., Wang, J. & Fan, K. Highly selective amorphous Ni-Cr-B catalyst in 2-ethylanthraquinone hydrogenation to 2-ethylanthrahydroquinone. *Chem. Commun.* 1236–1237 (2002).
